# The language disorder of prion disease is characteristic of a dynamic aphasia and is rarely an isolated clinical feature

**DOI:** 10.1371/journal.pone.0190818

**Published:** 2018-01-05

**Authors:** Diana Caine, Akin Nihat, Philippa Crabb, Peter Rudge, Lisa Cipolotti, John Collinge, Simon Mead

**Affiliations:** 1 NHS National Prion Clinic, National Hospital for Neurology and Neurosurgery (NHNN), University College London Hospitals NHS Foundation Trust, London, United Kingdom; 2 Department of Neuropsychology, NHNN, University College London Hospitals NHS Foundation Trust, London, United Kingdom; 3 MRC Prion Unit at UCL, UCL Institute of Prion Diseases, London, United Kingdom; University of Verona, ITALY

## Abstract

**Background:**

Akinetic mutism is a key diagnostic feature of prion diseases, however, their rapidly progressive nature makes detailed investigation of the language disorder in a large cohort extremely challenging. This study aims to position prion diseases in the nosology of language disorders and improve early clinical recognition.

**Methods:**

A systematic, prospective investigation of language disorders in a large cohort of patients diagnosed with prion diseases. 568 patients were included as a sub-study of the National Prion Monitoring Cohort. All patients had at least one assessment with the MRC Scale, a milestone-based functional scale with language and non-language components. Forty patients, with early symptoms and able to travel to the study site, were also administered a comprehensive battery of language tests (spontaneous speech, semantics, syntax, repetition, naming, comprehension and lexical retrieval under different conditions).

**Results:**

5/568 (0.9%) patients presented with leading language symptoms. Those with repeated measurements deteriorated at a slower rate in language compared to non-language milestones. Amongst the subgroup of 40 patients who underwent detailed language testing, only three tasks–semantic and phonemic fluency and sentence comprehension–were particularly vulnerable early in the disease. These tasks were highly correlated with performance on non-verbal executive tests. Patients were also impaired on a test of dynamic aphasia.

**Conclusion:**

These results provide evidence that the language disorder in prion disease is rarely an isolated clinical or cognitive feature. The language abnormality is indicative of a dynamic aphasia in the context of a prominent dysexecutive syndrome, similar to that seen in patients with the degenerative movement disorder progressive supranuclear palsy (PSP).

## Introduction

Recently there has been intense interest in language function in neurodegenerative diseases with the specific characteristics of language disorders now contributing significantly to the differential diagnosis. The primary progressive aphasias (PPA: fluent, non-fluent or logopenic) have been identified as a prominent and even pathognomonic feature in the cortical dementia syndromes of Alzheimer’s disease (AD) and fronto-temporal dementia (FTD) [1,2], while the conditions once characterised as ‘movement disorders’ (progressive supranuclear palsy [PSP], cortico-basal degeneration [CBD], amyotrophic lateral sclerosis [ALS], dementia with Lewy bodies [DLB]) are now also known to be associated with cognitive deficits that include language impairment [3]. Language symptoms have frequently been remarked upon, but relatively little studied in patients with prion disease (for example, [3–6]). The aim of the present study was to remedy this important gap by systematically characterising the language disorder in a large cohort of patients with prion disease.

Prion diseases are recognised as a heterogeneous group of transmissible neurodegenerative conditions which share a mechanism related to the templated misfolding of normal cell-surface prion protein into disease-associated multimeric forms [7]. They can be divided into three aetiological categories: inherited, as an autosomal dominant trait associated with mutations in the gene that encodes prion protein; acquired, through dietary or medical exposure to prions; or of unknown causes, termed sporadic disease. The most common form of human prion disease, sporadic Creutzfeldt-Jakob disease (sCJD), manifests as a rapidly progressive dementia associated with neurological signs like cerebellar ataxia, myoclonus and motor features, whereas inherited and acquired forms can be more slowly progressive. The rapid progression and advanced clinical state of most cases at presentation to specialist care makes detailed neuropsychological study difficult.

Although the pattern of cognitive impairment in prion disease has generally been considered pervasive, even patients with the rapidly progressive sporadic form of the disease have occasionally presented with focal cognitive deficits including aphasia [8–12]. Regardless of the timing of onset of language relative to other symptoms, prion patients are often described as becoming mute as the disease progresses (for example, [13]), and this clinical feature is enshrined as one of the cardinal diagnostic features of sporadic CJD (see http://www.cjd.ed.ac.uk/diagnosis-and-testing/diagnostic-criteria). It has recently been suggested that about 1% of patients with sCJD present with an isolated language disorder [6]. A study of two groups of familial prion patients found that as many as two thirds of patients with the 6-octapeptide repeat insertion (OPRI) mutation in the prion protein gene (*PRNP*) presented with impaired naming and apraxia as prominent features [14]. Two reports have raised the possibility of progressive non-fluent aphasia (PNFA) in the context of prion disease [15, 16], while the logopenic variant of PPA has also been identified in the context of [5].

In our previous investigation of cognitive function we demonstrated that while the leading cognitive symptom in most patients was executive dysfunction, even mildly affected patients were impaired on some language tasks, with verbal fluency and sentence comprehension particularly vulnerable [17]. Detailed investigation of language deficits in prion disease represents an opportunity to both deepen our understanding of the cognitive characteristics of this disease and, as already demonstrated in other neurodegenerative conditions, improve diagnostic acuity. Through the National Prion Monitoring Cohort, a UK-based prospective observational study of all types of prion disease [18], such a study in a relatively large group of patients has been possible for the first time. The aims of this project were threefold: (1) to investigate systematically the prevalence and characteristics of language impairment in an unselected series of patients with early stages of prion disease, (2) to evaluate the relationship between language impairment, and other clinical and cognitive symptoms (3) to situate the language disorder in prion disease in relation to that seen in other neurodegenerative diseases.

## Methods

### Participants

Participants were prospectively recruited to the National Prion Monitoring Cohort between October 2008 –April 2017, via the National Prion Clinic at the National Hospital for Neurology and Neurosurgery, London (NHNN). Ethics approval for the study was granted by the Eastern Multicentre Research Ethics Committee. Informed consent was provided by each subject or their next of kin. 568 patients were included, with the following diagnoses: sCJD (n = 427); inherited prion disease (IPD, n = 115); iatrogenic CJD (iCJD, n = 16); variant CJD (vCJD, n = 10). Diagnosis was established by prion protein gene sequencing, neuropathological examination, or using epidemiological diagnostic criteria [19].

### MRC Scale with verbal component

All patients had at least one assessment with the MRC Prion Disease Rating Scale (MRC Scale): a validated, functional outcome measure reflecting disease progression in CJD on a 20-point scale (where 20 is normal function). The MRC Scale is based on milestones in verbal (language), motor, continence, cognitive and social care domains [18]. The verbal component is a score of 0 (mute) to 4 (normal conversation), intended to identify gross onset and progression of language disturbance (Table 1).

**Table 1:** The verbal component of the MRC Scale [18].

Best Verbal Response	Descriptor
0	Mute
1	Incomprehensible sounds
2	Single words
3	Sentences but difficulty in findings words, uses incorrect words or is often disorientated / confused
4	Normal conversation

### Detailed language testing

A subset of 40 patients underwent a dedicated neuropsychological battery. This group, which includes those reported in our previous study of the overall cognitive profile of prion patients [17], comprised patients with sporadic, inherited or acquired prion disease [(40% female (n = 16), 60% male (n = 24); average age 51.4 years)] and 33 healthy control participants [(49% female (n = 16); 51% male (n = 17); average age 49 years)], recruited from patients’ families (if negative for a *PRNP* mutation, or not at risk). The patients were diagnosed as follows: inherited prion disease (IPD) (n = 26), including 5-OPRI (n = 4), 6-OPRI (n = 2), P102L (n = 10), D178N (n = 3), Q212P (n = 1), E200K (n = 3), A117V (n = 1), Y163X (n = 1) and E196K (n = 1) mutations; iCJD (n = 2); sporadic CJD (sCJD) (n = 11) or variant CJD (vCJD) (n = 1). Patients were included if they were definitely symptomatic but able to complete all the language tasks administered. Patients who were too impaired or who had an additional neurological disorder were excluded from this part of the study. Patients and controls were relatively matched for age (p = 0.45), gender (p = 0.61), and estimated full-scale IQ (p = 0.07) (see Table 2).

**Table 2:** Demographic information with estimated IQ based on the NART reading test for patients and controls, s.d. = standard deviation.

		Patients		Controls		
	N	Age Mean (s.d.)	IQ Mean (s.d.)	N	Age Mean (s.d.)	IQ Mean (s.d.)
Male	24	50.3 (9.4)	101.8 (16.6)	17	50.3 (12.5)	104.0 (11.2)
Female	16	52.9 (14.2)	102.4 (12.7)	16	47.4 (14.5)	108.0 (15.9)
Total	40	51.4 (11.4)	102.5 (15.1)	33	48.9 (13.4)	109.3 (11.7)

Assessment scores for patients with IPD recruited prior to developing symptoms, were taken from the first assessment following the point at which they were deemed symptomatic for the disease. Patients with other forms of the disease were always symptomatic at first presentation and the scores included here were those obtained at that time.

Some of the language tests administered comprised part of the general neuropsychological battery previously reported [17], but others were incorporated into this comprehensive language study designed specifically to address the characteristics of changes in language function in this disease. The additional tests were: *Spontaneous speech* (Cookie Jar Theft picture description, 60”); *Naming* to picture (Graded Naming Test, GNT [20]); *Semantics* (word definition, WAIS-III Vocabulary sub-test, Vocab [21]; concrete synonym matching; category fluency (Animals); *Syntax* (Test for Reception of Grammar, TROG [22]); *Repetition* (polysyllabic low-frequency words; non-words [23]; sentences); *Spelling* (Graded Difficulty Spelling Test, GDST [24]); *Executive language tasks* (letter fluency, [25]; Hayling Sentence Completion Test [26]; high/low constraint sentence completion test [27].

Four tests (word and sentence repetition, picture description and spelling) not found to be helpful in a preliminary analysis were excluded from the battery, to enable inclusion of more targeted tasks. The Hayling Sentence Completion Test and a test of sentence completion under different degrees of constraint were added to refine the language battery after initial analysis of language tasks. Only a subset of symptomatic patients (n = 14) were administered the latter tests, for which controls (n = 28) were at-risk, asymptomatic patients (gene positive or not tested and but without symptoms or signs on comprehensive neurological assessment). Additionally, the following non-language tests of executive function were administered: *Response inhibition* (Stroop Test [28]); *Cognitive flexibility* (Trail Making Test Part B [29]).

### Statistical analysis

Descriptive statistics (mean, median, interquartile range) for all patient MRC scores at each level of verbal component (0–4) were calculated (total patients n = 568, total individual assessments n = 2458, SPSS Statistics v22, IBM). A linear mixed modelling approach was used in 261 patients with sporadic CJD (1753 MRC Scale assessments) with 2 or more scores, to estimate linear rates of decline in verbal and non-verbal components of the MRC Scale (methodology as per [30]).

For the neuropsychological data, we used an independent t-test or its non-parametric equivalent to compare patients’ scores with those of controls for each test. To control for multiple comparisons, a p-value of <0.01 was always used as the criterion for significance. Missing data were treated with a missing at random approach. All statistical analyses were performed using the statistical package for the social sciences v.11.5 (SPSS, IBM, New York).

Following the procedure used in our previous study to assess the relative vulnerability of each language task, individual patient performance was compared with control mean scores and standard deviations. We calculated the percentage of patients whose performance was impaired on each task, and thus determined which language tasks were most vulnerable early in the disease. Finally, correlations between language tests and performance on tests of executive function were examined to assess for possible associations amongst tasks in these two cognitive domains.

## Results

### Onset of language dysfunction in the complete cohort

Only 5/568 (0.9%) patients (sCJD = 3, D178N = 1 MV, E196K = 1) presented with leading language symptoms (MRC Score = 19, verbal component = 3/4), with the following characteristics: sCJD age at onset (AAO) = 62/75/32yrs respectively, disease duration (DD) = 17/65 months/patient alive at 9 months, codon 129 = VV/MV/MM; D178N AAO = 38yrs, DD = 19 months, codon 129 = MV; E196K AAO = 68yrs, DD = 22 months, codon 129 = MM.

Most commonly, where language function was impaired at the first presentation, this was in tandem with other deficits. Contrary to the suggestion of early (more rapid) verbal impairment, using a linear mixed modelling approach in a subset of patients, we found a slightly faster rate of decline in *non-verbal* domains (-0.107 Δnon-verbal Scale/day, CI = 0.201–0.112) vs. verbal component scores (slope = -0.096 Δverbal Scale/day, CI = 0.091–0.101, p = 0.003). For patients with a verbal score of 3 (i.e. language impairment on the MRC Scale), the mean and median total MRC Scale scores were 11 (s.d +/- 3.90), indicating concurrent functional impairment in other domains (Table 3).

**Table 3:** MRC Scale and verbal component scores for all patients (n = 568).

Verbal Component Score	Mean MRC Scale Score (/20)	Median MRC Scale Score (/20)	Standard deviation	Range	Q1	Q3	IQR
4	15.6	16	3.7	5–20	13	19	6
3	11.0	11	3.9	3–19	8	14	6
2	5.9	5	2.7	2–17	4	7	3
1	2.9	3	1.8	1–14	2	4	2
0	0.9	0	1.3	0–9	0	2	2

### Detailed neuropsychological testing in a subset of patients and controls

On more detailed investigation of language in a smaller samples of patients (n = 40) analysed as a group (Table 4), and consistent with our previous findings, patients performed more poorly than controls on all language tests (p values ≤0.01), with one exception: concrete synonym matching (p = 0.056). We previously employed the heuristic that since those tests on which the largest proportion of patients were impaired were also those on which performance of the more mildly affected patients was affected, those were the tasks that could be considered most vulnerable to this disease. We therefore determined the percentage of patients impaired on each of the individual language tasks administered. Only a small number of the extensive range of language tasks employed were clearly more likely than others to be impacted early in the disease, and therefore to constitute a significant feature of the early presentation. Three tests stand out in this regard: the two verbal fluency tasks, category fluency (Animals: 72.5%) and letter fluency (FAS: 70.0%); and syntactic (sentence) comprehension (TROG: 60%). Just over half the patients had difficulty with object naming (GNT: 55%). Half or fewer of the patients were impaired on all other tasks, reflecting the overall heterogeneity of cognitive deficits once the illness is entrenched.

**Table 4:** Comparison of patients’ (n = 40) and controls’ (n = 33) mean scores on the battery of language tasks in a subset group that underwent detailed neuropsychological testing.

Test (Max. Score)	Patient Mean (s.d.)	Control Mean (s.d.)	T (df)	P (2-tailed)
Picture Description (no max)	101.9 (40.6)	131.0 (30)	-3.2(61)	.002
GNT (30)	18.6 (6)	22.7 (2.9)	-3.6(71)	.001
WAIS-III Vocab. (20)	8.8 (3.5)	11.5 (2.7)	-3.7(71)	.000
Synonym Matching (25)	20.8 (3.5)	22.2 (2.2)	-2(71)	.050
Animal fluency (no max)	12.3 (6.6)	22.6 (5.3)	-7.3(71)	.000
TROG (40)	33.2 (6.6)	38.5 (1.0)	-4.53(71)	.000
Word Repetition (40)*	39.4 (1.0)	40.0(.0)	165.0/-5.5	.000
Non-word Repetition (20)	15.3 (5.0)	18.8 (1.8)	-3.8(71)	.000
Sentence Repetition (15)*	27. (2.7)	30.00 (.0)	264.0/-4.4	.000
GDST (30)	14.6 (9.66)	20.97 (5.60)	-3.24(61)	.002
FAS (no max)	26.60 (15.37)	44.36 (12.89)	-5.28(71)	.000
Stroop test (112)	55.28 (32.89)	103.09 (8.13)	5. (71)	.000
Trail Making B (no max)	158.08 (108.29)	60.73 (22.04)	5.13(71)	.000

*Mann-Whitney U/Z

Patients produced reduced output relative to controls, demonstrated in their performance on fluency tasks and spoken picture description (CJT: 53.3%) On the picture description test, patients produced fewer words altogether (p = .006) and proportionately fewer nouns (p = .005), while the difference also approached significance with respect to verbs (p = .028). There was no difference between patients’ and controls’ performance on the closed word class of pronouns proportionate to the total of nouns and pronouns produced (p = .357), or the number of pauses (p = 0.203).

The characteristics of the three language tests most vulnerable to prion disease [31, 32]) together with the previous observation that executive dysfunction is the leading cognitive symptom in this disorder [17] raised the question of whether the executive demands of these particular tasks were responsible for their vulnerability to impairment in prion patients. We therefore examined the relationship between performance on these three tasks, together with the naming test, with scores on two non-verbal executive tasks (Stroop test, Trail Making Test Part B). We found strong correlation between the Animal fluency, FAS, and TROG tasks and the non-verbal executive tests (r = 0.46–0.73, all p<0.01). In contrast, the GNT was strongly correlated with each of the other language tasks but not the non-verbal executive tests (r = 0.17–0.25, all p>0.05).

Finally, the strongly executive flavour to the neuropsychology profiles in general, and the executive demands of the language tests on which patients seemed to be most affected, led us to ask whether prion disease might be associated with a dynamic aphasia such as has been identified in patients with PSP [27, 33]. A subset of patients was therefore administered both the Hayling Sentence Completion Test, a recognised test of executive function [34]; and following Robinson et al. [27], a second test of sentence completion under varied conditions of contextual constraint (called here the high/low completion task). The Hayling Test has two conditions: first, the target word is one that meaningfully completes the sentence and is strongly suggested by the sentence (e.g. ‘he posted the letter without a…’); second, the sentence still strongly suggests a response but the target is now a word that is completely unrelated to the sentence (e.g. ‘the captain wanted to stay with the sinking….GREEN’). The second condition therefore demands a degree of response inhibition for successful execution. In the high/low test of sentence completion the target word in each case is meaningful but in the first condition it is highly constrained by the sentence (e.g. ‘to pay for the tickets he wrote a…’), while in the second condition the constraint is much lower (e.g. ‘she had never known anyone so…’). The key characteristic of dynamic aphasia patients is that they are able to perform most language tasks, including highly constrained sentence completion tasks, satisfactorily but that they fail precisely in the condition in which they are required to produce words under levels of low constraint, that is, where there are numerous response options.

T-tests were performed to compare patients with controls’ scores in the four conditions of these two tasks. As can be seen in Table 5 the symptomatic patients were slightly slower than the asymptomatic controls on all four timed tasks but this difference was not significant except on the test of sentence completion under low constraint.

**Table 5:** Comparison of patients (n = 40) and controls’ (n = 33) performance on the Hayling and High/Low sentence completion tasks in a subset group that underwent detailed neuropsychological testing.

Test	Patient (n = 14) Mean (s.d.)	Control (n = 26) Mean (s.d.)	T (df)	p-value
Hayling Test connected completion: time	8.79 (7.34)	4.96 (4.29)	2.09 (38)	.043
Hayling Test unconnected completion: time	39.31 (39.76)	33.35 (34.46)	.48 (37)	.631
Hayling Test unconnected completion: errors	19.00 (26.59)	4.62 (6.78)	2.62 (37)	.013
High/Low completion task—high constraint: time	5.43 (5.80)	2.65 (2.62)	2.09 (38)	.043
High/Low completion task- low constraint: time	68.79 (42.22)	36.85 (22.24)	3.15 (38)	**.003**

## Discussion

This study aimed to thoroughly examine the language disorder frequently observed in prion disease, which is a component of well-established epidemiological criteria. We subjected a large, mixed population of prion disease patients to comprehensive assessment of speech and language skills, in addition to both the short cognitive examination and the full neuropsychological battery on which we previously reported [17].

We took advantage of the repeated measures of the MRC Scale and its language component to address the question of the time point at which language problems assert themselves relative to the overall disease course, and rates of change of verbal and non-verbal components of functional assessments. We found <1% of patients in whom language was a leading symptom, or who might be diagnosed with a primary progressive aphasia. In general, language became compromised alongside abnormalities of other cognitive and neurological domains, but contrary to previous reports, deteriorated at a *slower rate*.

We previously demonstrated that although the cognitive effects of prion disease are pervasive, early disease is characterised by prominent executive impairment, parietal dysfunction, and a largely expressive dysphasia [17]. In the present study we sought comprehensively to investigate all aspects of language function in this disease. Our results show that while, indeed, all aspects of language *can* become compromised in the context of more severe disease, it is just two specific aspects of language processing that are liable to be vulnerable early in the disease course: verbal fluency (Letter and category) and sentence comprehension. None of the other language tasks, from our comprehensive battery including repetition, semantics, naming and spontaneous speech production, were shown to be vulnerable to the same extent early in the milder stages of the disease.

The present findings demonstrate clearly that, in general, prion patients do not display the cardinal signs of the three primary progressive aphasias—neither the semantic decline of progressive fluent aphasia, the phonological problems and speech apraxia central to PNFA, nor the hallmark dissociation between word and sentence repetition of logopenic aphasia. There is evidence of mildly reduced sentence and non-word repetition in some patients, at least some of which is likely accounted for by the presence of dysarthria. At the same time, the fact that almost half of the patients were impaired on all the language tasks confirms both the clinical impression and numerous reports suggesting that speech and language deficits are a common feature of the disease.

The verbal fluency and sentence comprehension tasks on which the patients perform most poorly are usually considered to be distinct from one another in their cognitive underpinnings: phonemic versus semantic fluency; spoken production versus comprehension. However, studies examining their respective task demands clearly demonstrate a striking overlap in their call on intact working memory for effective execution in each case [31, 32].

To further explore the speculation that these language deficits reflect an executive rather than a phonological provenance, we examined and found strong correlations between these three language tasks in question and two non-language based executive tests, the Stroop test and the Trail Making Test Part B. At the same time, the GNT was shown to correlate with the language but not the executive tests. While not conclusive, this pattern of correlations is supportive of the idea that the patients’ primary executive deficits may indeed be contributing to their poor performance on these tasks. This was further borne out in the observation that symptomatic patients tended to produce more errors than those who are asymptomatic but at risk on the unconnected condition of the Hayling Sentence completion task, a test requiring inhibition of a highly constrained response. It is important to note that the patients performed just as well as those at risk on the connected condition, indicating that it was not responding per se or response speed that was an issue.

We further sought to address the question of whether the patients’ language problems might fall under the umbrella of a dynamic aphasia. Indeed, we found that patients performed poorly compared to controls on a test of word generation under conditions of low versus high contextual constraint. Again, it is important that there was no difference in the high constraint condition; patients’ performance suffered precisely in the condition in which word selection was less determined by context, consistent with the pattern in patients identified as having dynamic aphasia [35].

In our previous study, we suggested that amongst neurodegenerative diseases, prion disease resembles movement disorders with associated dementia syndromes including corticobasal degeneration (CBD), PSP, Amyotrophic lateral sclerosis (ALS), and perhaps Lewy body disease. The present findings further support that view, and the combination of executive deficits and dynamic aphasia we have identified in prion disease bears comparison especially with the cognitive profile of PSP [35, 36]. While there has been no large group study of language specifically in PSP, dynamic aphasia has been more often associated with that pathology than with any other neurodegenerative disorder [37]. It should be noted that although this was our overall finding in a large group, it does not preclude the possibility of alternative language disorders in rare patients.

In summary, this study represents the largest and most comprehensive investigation of speech and language function and decline in prion disease yet undertaken. Using a combination of group and individual analyses, we have demonstrated that prion disease rarely presents as a primary progressive aphasia, nor can the language problems in prion disease be characterised in terms of the leading features of any of the three typical PPA’s. Rather, speech and language decline is highly correlated with executive deficits, so performance is affected on tasks calling most strongly on executive function and working memory–verbal fluencies and syntactic comprehension. This includes speech production under conditions of low contextual constraint characteristic of a dynamic aphasia. The present findings thus further confirm our previous observation that prion disease is properly considered within the constellation of degenerative movement disorders with a cognitive and language profile most like that seen in PSP. It points firmly to the appropriate language tests for optimal early detection of the disease.

## Supporting information

S1 File Full cohort MRC scores. Total MRC Scale scores for all patients (n = 568).
(XLSX)

S2 File Full neuropsychometry scores. Neuropsychometry assessment scores for 40 CJD patients and 33 controls. Abbreviations: *gnt* (Graded naming test); *cjt* (Cookie jar theft picture description); *vocab* (WAIS-III vocabulary sub-test); *concrete_syn* (Concrete synonym matching); *gdst* (Graded difficulty spelling test); *trog* (Test for reception of grammar); *rep_non_word* (Non-word repetition); *rep_low_word* (Low-frequency word repetition); *fas* (Verbal fluency); *animals* (Category fluency); *tmt_b* (Trail-making test); *stroop* (Stroop test).
(SAV)

S3 File Extended language test scores. Additional language test (Hayling sentence completion and sentence completion under different degrees of constraint) for 14 patients with CJD and 28 controls.
(SAV)
